# Family Characteristics as Risk Factors for Childhood Acute Lymphoblastic Leukemia: A Population-Based Case-Control Study

**DOI:** 10.1371/journal.pone.0013156

**Published:** 2010-10-04

**Authors:** Martin Feller, Martin Adam, Marcel Zwahlen, Pierluigi Brazzola, Felix Niggli, Claudia Kuehni, for the Swiss Pediatric Oncology Group (SPOG) and the Swiss National Cohort (SNC)

**Affiliations:** 1 Institute of Social and Preventive Medicine, University of Bern, Bern, Switzerland; 2 Swiss Tropical and Public Health Institute, Department of Epidemiology and Public Health, Basel, Switzerland; 3 University of Basel, Basel, Switzerland; 4 Ospedale Regionale di Bellinzona e Valli–Bellinzona, Bellinzona, Switzerland; 5 Pediatric Oncology Unit, University Children's Hospital Zürich, Zürich, Switzerland; Aga Khan University, Pakistan

## Abstract

**Background:**

To date, few risk factors for childhood acute lymphoblastic leukemia (ALL) have been confirmed and the scientific literature is full of controversial “evidence.” We examined if family characteristics, particularly maternal and paternal age and number of older siblings, were risk factors for childhood acute lymphoblastic leukemia (ALL).

**Methodology/Principal Findings:**

In this population-based nationwide matched case-control study, patients 0–14 years of age with ALL diagnosed 1991–2006 and registered in the Swiss Childhood Cancer Registry were linked with their census records of 1990 and 2000. Eight controls per case were selected from the census. The association between family characteristics and ALL was analyzed by conditional logistic regressions. We found that increasing maternal age was associated with incidence of ALL in the offspring (OR per 5-year increase in maternal age 1.18, 95% CI 1.05–1.31; p = 0.004), remaining stable (trend OR 1.14, 95% CI 0.99–1.31; p = 0.060) after adjustment for other risk factors. The association with paternal age was weaker (OR per 5-year increase 1.14, 95% CI 1.01–1.28, p = 0.032) and disappeared after adjustments. Number of older siblings was not associated with risk of ALL in the overall group of children aged 0–14 years at diagnosis. However, we found a negative trend between number of older siblings and ALL diagnosed at age 0–4 years (OR per sibling 0.85, 95% CI 0.68–1.06; p = 0.141) and a positive trend for ALL diagnosed at age 5–9 (OR 1.34, 95% CI 1.05–1.72; p = 0.019), with some evidence for an effect modification (p-value for interaction  = 0.040).

**Conclusions:**

As in other studies, increasing maternal, but not paternal age was associated with risk of ALL. We found only a weak association with the number of older siblings, suggesting a delay in disease manifestation rather than a decrease in incidence.

## Introduction

Studies on the associations between family characteristics and incidence of childhood acute lymphoblastic leukemia (ALL) have shown discrepant results [1], [2], [3], [4], [5], [6], [7], [8], [9], [10]. While most studies found a positive association between maternal age at birth and ALL [1], [4], [5], [6], [7], others did not [8], [9], [11]. Data on very young mothers (<20 years) are particularly controversial [10], [12], and it is unclear whether an association with paternal age exists [4], [7], [11], [13], [14], partly because paternal and maternal age are correlated and their effects not easy to disentangle. Some publications suggested that the association between paternal age and risk of ALL disappears after adjusting for maternal age and birth order [7], [13], [14].

The association between birth order and ALL has been debated for a long time. Greaves' delayed-infection hypothesis, originally published for ALL in 2–5 year olds [15], [16], [17], suggests that children with older siblings, who have contact with infectious agents already in infancy are less likely to develop ALL than first born or only children. While some studies reported a decreasing risk of ALL with increasing birth order [2], [4], [9], others did not confirm this observation [6], [8], [13]. Only few studies analyzed their results stratified by age at diagnosis or subtype of ALL, which might be important in relation to causal mechanisms [18].

The framework of an ongoing nationwide study linking the Swiss Childhood Cancer Registry (SCCR) with census records [19] offered an ideal opportunity to study these questions. Using a matched case-control design to minimize selection bias, participation bias and recall bias, we determined whether family characteristics, particularly parental age and number of older siblings, were associated with incidence of childhood ALL. In addition, we assessed whether the strength of this associations varied by age at diagnosis.

## Methods

### Study design and study populations

This study was based on combined population-based data, obtained by linking the Swiss Childhood Cancer Registry (SCCR) with census records. Information on exposures (family characteristics) came from the census, while information on disease was obtained from the SCCR. The Swiss Childhood Cancer Registry (SCCR, www.childhoodcancerregistry.ch) [20] records all children diagnosed with cancer (according to the International Classification of Childhood Cancer, third revision (ICCC3) [21]) in Switzerland since 1976. For this study, we included all children diagnosed with ALL at age 0–14 years between 1991–2006, who were resident in Switzerland at the time of diagnosis and were recorded either in the 1990 or 2000 census.

Information on control children was retrieved from the census datasets of 1990 and 2000, which include detailed but anonymized information on family characteristics and socio-economic determinants for every citizen in Switzerland [19]. Because routine data in Switzerland are anonymous and there is no personal identifier to link datasets, we had to link the cases from the SCCR to the census records using a probabilistic linkage procedure. Information contained in both datasets (sex, date of birth and place of residence) were used for linking the records. Only cases born before, and diagnosed after a census were included in this study, because we wanted to avoid selection bias related to selective exclusion of children who had died before the census and thus could not have been registered in the census (for instance a child born in 1996 who died in 1999). For cases (and their matching controls) who were born before the 1990 census and diagnosed after the 2000 census, we used the census data from the 2000 census. For every case, up to eight control children individually matched for year of birth and sex were randomly selected from the census.

### Exposures

We examined the following family characteristics available from the census for their association with incidence of ALL: maternal and paternal age at birth of the child (in four categories: <25, 25–29, 30–34 and ≥35 years), and number of older children living in the same household as the study participants (0, 1 or ≥2), respectively. This relates mostly to siblings, but might include a small proportion of non-related children living in the same household (e.g. children of a step-parent). To not overburden our terminology, we will call them older siblings throughout this paper for simplicity. Younger siblings were deliberately excluded from the analysis: being born on average 2–4 years later than the index child, they cannot have had the hypothesized protective effect during the index child's infancy. As a potential confounder we included the highest attained educational level of the father or the mother (whoever attained the higher education) as a proxy measure for the socio-economic status of the family. We used three categories: “primary education” (up to 9 years of education), “secondary education” (10 to 16 years, high school, teachers training colleges, technical colleges and upper vocational education) and “tertiary education” (16 years or more).

### Statistical analysis

We descriptively show percentage distributions of characteristics of the cases and controls. To account for the matched design, we fitted univariable conditional logistic regression models to assess associations between each variable of interest and ALL. Associations are presented as odds ratios (OR) with 95% confidence intervals, with an OR>1 indicating an increased risk for ALL. All variables of interest were included in the model by using indicator variables for categories or included continuously to assess trends. As a next step, we fitted multivariable conditional logistic regression models including all variables of interest. Finally, because Greaves' delayed-infection hypothesis was originally published for young children [16], we stratified the dataset into age groups 0–4, 5–9 and 10–14 years at diagnosis and assessed separately for these age groups the crude and adjusted odds ratios between ALL and number of older siblings. A stronger protective effect in the youngest children with decreasing effect in older age groups would support Greaves' delayed-infection hypothesis. We tested this effect modification by age formally, using likelihood ratio tests. As a sensitivity analysis, calculations were repeated including only children with precursor B-cell ALL.

All p-values were two-sided with a p-value of ≤0.05 indicating statistical significance. Statistical analyses were performed in STATA (Version 11.0, Stata Corporation, College Station, Texas, USA).

### Ethical approval

The Swiss Childhood Cancer Registry obtained an exemption from the Swiss Expert Commission for Professional Secrecy in Medical Research which allows nationwide collection and analysis of non-anonymized data. Parents are granted the right to refuse that the data on their children is included in the SCCR. The data of the Swiss National Cohort is fully anonymous and approval for the design and conduct of this project was given by the Swiss Federal Statistical Office via a specific legal contract.

## Results

Overall, 716 eligible children with ALL, resident in Switzerland and diagnosed between 1991 and 2006 at an age of 0–14 years were registered in the SCCR. Of these, 426 cases met the inclusion criteria for a probabilistic record linkage with the data of the 1990 or 2000 census. We successfully linked 425 (99.8%) to the census and selected 3′350 sex- and age-matched controls. Of the cases, 59% were males, 80% Swiss Nationals, and 76%, 21% and 3% lived in the German, French or Italian part of Switzerland, respectively (Table 1). Leukemia had been diagnosed at the age of 0–4 years in 175 (41%), at the age of 5–9 in 130 (31%) and at the age of 10–14 years in 120 (28%, Table 1). This includes a relatively low proportion of children diagnosed at age 0–4, because this age group was least likely to satisfy the study inclusion criteria (born before and diagnosed after a census). There were 334 (78.6%) cases with precursor B-cell lymphoblastic leukemia among the 425 cases (Table 1).

**Table 1:** Baseline characteristics of cases with ALL (n = 425) and control children (n = 3350).

	Cases		Controls a	
Characteristics	Number	%	Number	%
**Gender** a				
male	250	58.8	1961	58.5
female	175	41.2	1389	41.5
**Nationality**				
Swiss	341	80.2	2629	78.5
Non-Swiss	84	19.8	721	21.5
**Language region**				
Swiss German	324	76.2	2447	73.0
Swiss French	90	21.2	794	23.7
Swiss Italian	11	2.6	109	3.3
**Age at census [years]** a				
0–4	253	59.5	1998	59.6
5–9	119	28.0	944	28.2
10–14	53	12.5	408	12.2
**Age at diagnosis [years]**				
0–4	175	41.2	-	-
5–9	130	30.6	-	-
10–14	120	28.2	-	-
**Immunophenotype (ICD-O-3)**				
Precursor B-cell lymphoblastic leukemia	334	78.6	-	-
Precursor T-cell lymphoblastic leukemia	61	14.4	-	-
Precursor cell lymphoblastic leukemia NOS	14	3.3	-	-
Burkitt cell leukemia	12	2.8	-	-
Others b	4	0.9	-	-

a controls were matched on age and sex.

b 1 precursor T-cell lymphoblastic lymphoma, 1 acute biphenotypic leukemia, 1 lymphoid leukemia NOS, 1 aggressive NK-cell leukemia.

In the overall sample of 0–14 year olds we found evidence for an association between maternal age at delivery and ALL in the child, with increasing risks for increasing maternal age. Compared to children whose mothers were aged <25 at delivery, odds ratios for ALL were 1.14, 1.38 and 1.58 for children whose mothers were 25–29, 30–34 and >34, respectively (p for trend  = 0.004). This resulted in an OR of 1.18 (95% CI 1.05–1.31) per 5-year increase of maternal age. Because of conflicting results for very young mothers in previous publications, we also looked at very young mothers aged <20 years at birth (11 cases, 65 controls). We found a trend for a higher risk of ALL in their offspring (OR 1.77, 95% CI 0.88–3.56) compared to children whose mothers were aged 20–24 at birth (p = 0.112).

Association with paternal age was less clear, suggesting rather an u-shaped association with the lowest risk in children of fathers aged 25–29 at birth of the child, and higher risks in offspring of younger and older fathers (p value for categorical analysis  = 0.044, p for linear trend across age groups  = 0.032; Table 2). There was no association between socio-economic status and risk of ALL (p value for categorical analysis  = 0.838).

**Table 2:** Associations between family characteristics and ALL in Swiss children aged 0 to 14 years, unadjusted.

		Cases		Controls a		Odds ratio^b^	95% CI b	p trend c
		(n = 425)		(n = 3350)				
		Number	%	Number	%			
**Number of older siblings**								
	0 older siblings	206	48.5	1632	48.7	1.00	-	
	1 older sibling	158	37.2	1221	36.5	1.03	0.83 to 1.29	0.999
	≥2 older siblings	61	14.3	497	14.8	0.98	0.72 to 1.33	
**Maternal age at birth [years]**								
	<25	70	16.5	672	20.1	1.00	-	
	25–29	154	36.2	1308	39.0	1.14	0.84 to 1.54	
	30–34	135	31.8	950	28.4	1.38	1.02 to 1.89	0.004
	≥35	64	15.1	396	11.8	1.58	1.10 to 2.29	
	missing	2	0.5	24	0.7			
**Paternal age at birth [years]**								
	<25	32	7.5	234	7.0	1.00	-	
	25–29	100	23.5	976	29.1	0.75	0.49 to 1.14	
	30–34	138	32.5	1125	33.6	0.90	0.59 to 1.35	0.032
	≥35	130	30.6	831	24.8	1.15	0.76 to 1.74	
	missing	25	5.9	184	5.5			
**Socio-economic status** d								
	primary	89	20.9	736	22.0	1.00		
	secondary	214	50.4	1647	49.1	1.08	0.83 to 1.41	0.773
	tertiary	122	28.7	967	28.9	1.05	0.79 to 1.40	

a controls were individually matched to cases on sex and age.

b using a univariable conditional logistic regression model.

c p values for trend were obtained from a conditional logistic regression including the variable continuously, for number of older siblings, the coding was 0, 1, 2, for socio-economic status, the coding was 1, 2, 3.

d socio-economic status was defined as the highest education attained by either the father or the mother.

**Figure 1:**
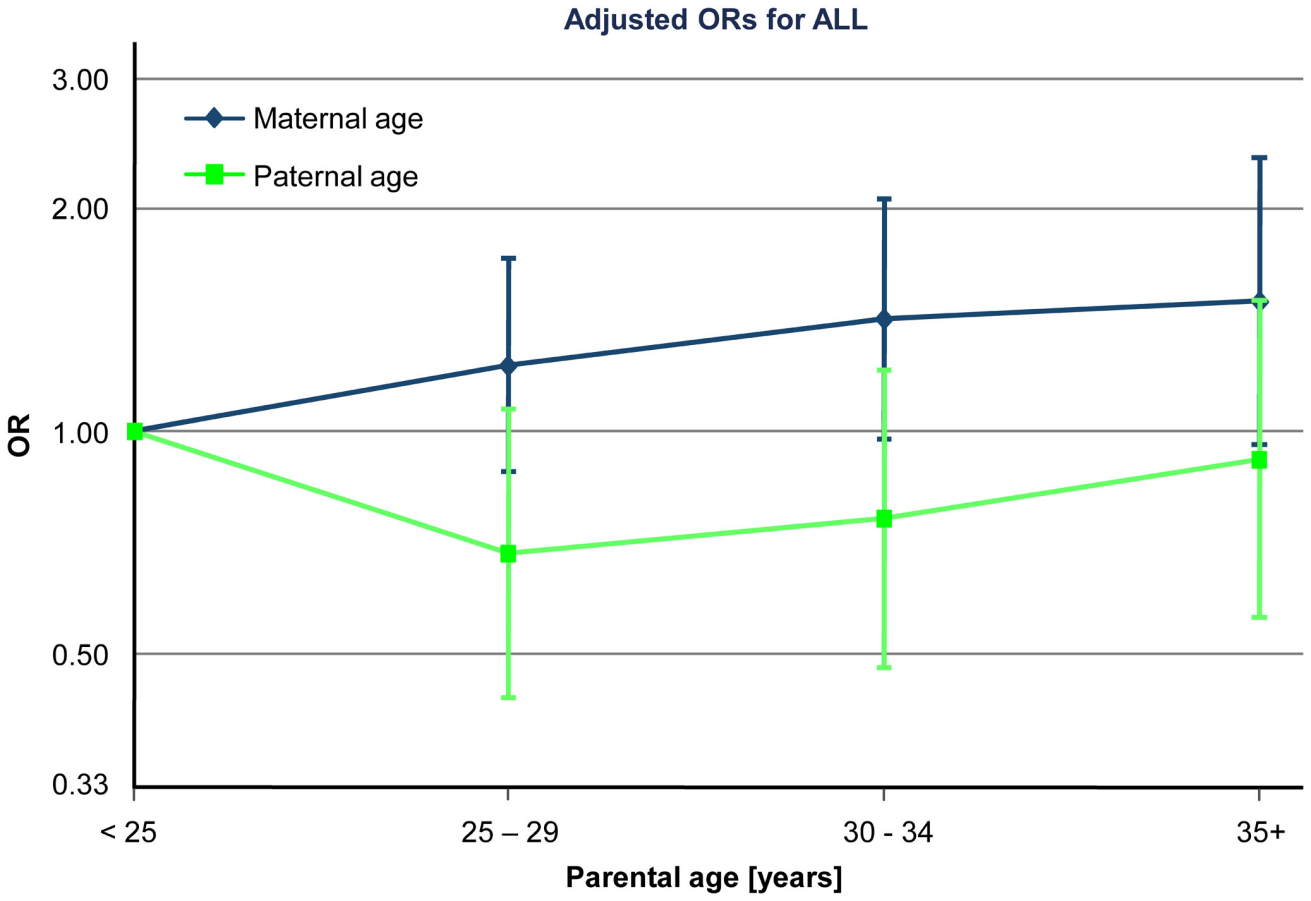
Parental age and ALL. Adjusted odds ratios for ALL comparing children with older mothers and fathers to children whose parents were aged less than 25 years. All odds ratios are adjusted for maternal and paternal age, respectively, and number of older siblings and socio-economic status. The analyses are based on 425 cases and 3′350 controls, aged 0–14 years. 11 cases (65 controls) were born to mothers younger than 20 years and 3 cases (21 controls) were born to fathers younger than 20 years. Figure footnote: y-axis is log-scaled.

In the multivariable analysis, adjusting simultaneously for maternal and paternal age, socio-economic status and number of older siblings (Figure 1), maternal age remained linearly associated with ALL (p = 0.060) but not paternal age (p = 0.672).

Number of older siblings was not associated with risk of ALL in the overall age group of 0–14 year olds (Table 2), with odds ratios for ALL of 1.03 and 0.98 when comparing children with 1 and more older siblings respectively to the reference group of children without older siblings (p for trend  = 0.999). Results remained similar when adjusting for the other exposures. However, there was some evidence for effect modification by the age group of the child at diagnosis (p value of the likelihood ratio test for interaction between number of siblings and age at diagnosis  = 0.040). When we stratified the analysis by age group at diagnosis (Table 3), we found a trend for a negative association between number of older siblings and ALL in children diagnosed at the age of 0–4 years, with an odds ratio of 0.86 and 0.72 in children with 1 or more older siblings compared to those without (trend OR per 1 unit increase in birth order: 0.85, 95% CI 0.68–1.06; p = 0.141). In children diagnosed at the age of 5–9 years there was some evidence for an opposite trend with increased odds ratios of 1.26 and 1.84 for children with one and more older siblings compared to those without (trend OR 1.34, 95% CI 1.05–1.72; p = 0.019). For children diagnosed after the age of 9 years, there was no clear association between ALL and number of older siblings (p = 0.521). Results remained essentially similar after adjustment for the other exposures (Table 3, second column). For maternal and paternal age, we found no evidence for effect modification by age at diagnosis (p value for interaction with maternal and paternal age 0.717 and 0.310, respectively). When we restricted the analysis to cases with precursor B-cell ALL and their matching controls (334 cases, 2638 controls), all results remained similar (data not shown).

**Table 3:** Crude and adjusted odds ratios (OR) for ALL comparing children with older siblings to children without older sibling, stratified by age groups.

		Crude OR a	95% CI a	p a	p trend e	Adjusted OR f	95% CI f	p f	p trend e
**0–4 year old children** b									
	0 older siblings	1.00	-	-		1.00	-	-	
	1 older sibling	0.86	0.61 to 1.21	0.393	0.141	0.83	0.58 to 1.19	0.310	0.054
	≥2 older siblings	0.72	0.45 to 1.14	0.161		0.63	0.38 to 1.02	0.059	
**5–9 year old children** c									
	0 older siblings	1.00	-	-		1.00	-	-	
	1 older sibling	1.26	0.84 to 1.89	0.268	0.019	1.21	0.78 to 1.86	0.391	0.090
	≥2 older siblings	1.84	1.12 to 3.03	0.017		1.61	0.94 to 2.78	0.085	
**10–14 year old children** d									
	0 older siblings	1.00	-	-		1.00	-	-	
	1 older sibling	1.12	0.74 to 1.69	0.586	0.521	1.11	0.73 to 1.68	0.636	0.379
	≥2 older siblings	0.62	0.29 to 1.33	0.220		0.55	0.25 to 1.22	0.141	

a univariable conditional logistic regression model.

b 175 cases, 1382 controls.

c 130 cases, 1032 controls.

d 120 cases, 936 controls.

e p values for trend were obtained including the variable continuously; the coding for number of older siblings was 0, 1, 2.

f multivariable conditional logistic regression adjusted for maternal age, paternal age and socio-economic status.

## Discussion

This nationwide study used linked records from the Swiss Childhood Cancer Registry and census records [22], to study the association between family characteristics and risk of ALL in children. It found evidence for an increasing risk of ALL with rising maternal age, while there was no clear pattern for paternal age, particularly after adjusting for potential confounders. Looking at the whole group of children diagnosed with ALL between age 0 and 14 years, there was no association with number of older siblings. However, we found evidence for an effect modification by age at diagnosis, with a trend for a protective effect of siblings in 0–4 year olds, while 5–9 year olds with older siblings had an increased risk of ALL. This might be compatible with a delayed development, rather than a protection from ALL for children with older siblings.

This study had strengths and weaknesses. By linking records from the national childhood cancer registry and the Swiss census and avoiding direct contact with cases and controls, our study was based on a representative sample from the entire population of Switzerland, and we used a study design that minimizes selection bias due to selection of non-representative controls. Also all information on exposures was derived from routinely collected census records, assessed before diagnosis and in a similar way for cases and for controls. Information on cancer diagnosis was derived from the Swiss Childhood Cancer Registry.

A limitation of this study was the relatively low number of cases we could use (n = 425), resulting in limited statistical power to detect small to moderate associations, and to investigate effect modifications by age at diagnosis and diagnostic subgroups. Also, we did not have information on additional potential confounders such as birth weight, gestational age and nursery care. Further, for a few children (those aged 14–15 years at diagnosis, and diagnosed shortly after a census) the number of older siblings included in our analysis might have been slightly underestimated, because some older siblings could have already moved out of the household at the time of the census. However, this can only have affected very few if any families, because in Switzerland most children live with their parents at least until age 20.

Our results for maternal age at delivery are in line with previous studies which suggested that high maternal age but not paternal age at delivery were risk factors for ALL [7], [13], [14]. However there is still controversy if the relationship between maternal age at delivery and ALL is linear or u-shaped as some studies observed an increased risk of ALL in children of mothers aged <20 years [2], [4], [12], [23]. In an exploratory analysis, our data was compatible with a higher risk of ALL in children of mothers aged <20 years at delivery although a chance finding cannot be excluded.

As to number of older siblings, a proxy factor for early exposure to common childhood infections, we found no evidence for an association with ALL in the whole age group, in accordance with some [1], [8], [11], [13], [14], [24] but not all previous work [4], [9]. One explanation for the discrepancies between studies could be that birth order is an insufficient proxy for infections in infancy in some settings but adequate in others. Another reason for differing results might be the different proportions of children diagnosed at different age in the published studies. When we analyzed our data stratified for age, we observed a weak negative association between number of older siblings and ALL in 0–4 year old children, as did two previous studies [2], [4]. In 5–9 year old children we found an opposite trend: children with older siblings in this age group had an increased risk for ALL. An explanation for this could be that infections in infancy do only delay but not prevent the progress to overt ALL. We had a relatively small sample size with limited statistical power. Therefore this hypothesis needs to be further tested. To our knowledge no other studies have analyzed the association between birth order and ALL stratified by age. Future studies should also try to include better markers for infections in infancy (e.g. number of viral colds), ideally measured prospectively. A promising approach could be aggregated prospective cohort studies as exemplified by the International Childhood Cancer Cohort Consortium (I4C).[25]
